# New targets acquired: Improving locus recovery from the Angiosperms353 probe set

**DOI:** 10.1002/aps3.11420

**Published:** 2021-06-14

**Authors:** Todd G. B. McLay, Joanne L. Birch, Bee F. Gunn, Weixuan Ning, Jennifer A. Tate, Lars Nauheimer, Elizabeth M. Joyce, Lalita Simpson, Alexander N. Schmidt‐Lebuhn, William J. Baker, Félix Forest, Chris J. Jackson

**Affiliations:** ^1^ National Herbarium of Victoria Royal Botanic Gardens Victoria Melbourne Australia; ^2^ School of Biosciences University of Melbourne Melbourne Australia; ^3^ Centre for Australian National Biodiversity Research CSIRO Canberra Australia; ^4^ School of Fundamental Sciences Massey University Palmerston North New Zealand; ^5^ James Cook University Cairns Australia; ^6^ Australian Tropical Herbarium James Cook University Cairns Australia; ^7^ Royal Botanic Gardens, Kew Richmond Surrey TW9 3AE United Kingdom

**Keywords:** Angiosperms353, HybPiper, locus recovery, target capture, target file

## Abstract

**PREMISE:**

Universal target enrichment kits maximize utility across wide evolutionary breadth while minimizing the number of baits required to create a cost‐efficient kit. The Angiosperms353 kit has been successfully used to capture loci throughout the angiosperms, but the default target reference file includes sequence information from only 6–18 taxa per locus. Consequently, reads sequenced from on‐target DNA molecules may fail to map to references, resulting in fewer on‐target reads for assembly, and reducing locus recovery.

**METHODS:**

We expanded the Angiosperms353 target file, incorporating sequences from 566 transcriptomes to produce a ‘mega353’ target file, with each locus represented by 17–373 taxa. This mega353 file is a drop‐in replacement for the original Angiosperms353 file in HybPiper analyses. We provide tools to subsample the file based on user‐selected taxon groups, and to incorporate other transcriptome or protein‐coding gene data sets.

**RESULTS:**

Compared to the default Angiosperms353 file, the mega353 file increased the percentage of on‐target reads by an average of 32%, increased locus recovery at 75% length by 49%, and increased the total length of the concatenated loci by 29%.

**DISCUSSION:**

Increasing the phylogenetic density of the target reference file results in improved recovery of target capture loci. The mega353 file and associated scripts are available at: https://github.com/chrisjackson‐pellicle/NewTargets.

Target enrichment (also known as target capture, exon capture, or Hyb‐Seq) has become the leading high‐throughput sequencing methodology for phylogenomics, offering reliable retrieval of hundreds or thousands of loci at a reasonable price per base pair (Cronn et al., 2012; Grover et al., 2012; Barrett et al., 2016; Bragg et al., 2016; Dodsworth et al., 2019). The method has proven useful for resolving relationships at all taxonomic ranks, including higher‐level phylogenetic relationships among orders or families, as well as lower‐level relationships among genera or species, and for species delimitation (Bi et al., 2013; Nicholls et al., 2015; Song et al., 2017; Choi et al., 2019; Breinholt et al., 2021). Target enrichment uses available genome sequence information in the form of genomes, transcriptomes, or genome skimming data to identify a set of target loci (e.g., genes, exons, or ultra‐conserved elements [UCEs]), which are typically low‐ or single‐copy (Faircloth, 2017; McKain et al., 2018). From the target loci set, short 80–120 bp RNA baits (also called probes) are designed, to create a “bait kit.” These short RNA baits are used in a hybridization reaction to bind to DNA fragments matching the target loci, which are then captured and PCR‐amplified for sequencing. The increasing availability of genomic resources held in public repositories, combined with the use of pipelines to identify low‐ or single‐copy genes based on these resources, has enabled bait kit design for a wide range of plant groups (Kadlec et al., 2017; Campana, 2018; Chafin et al., 2018; Vatanparast et al., 2018).

Universal bait kits, such as the Angiosperms353 bait kit, aim to capture the same set of loci from samples representing significant phylogenetic breadth and evolutionary timescales (Bossert and Danforth, 2018; Breinholt et al., 2021; Johnson et al., 2019). Such kits typically require a larger number of baits to encompass the sequence diversity potentially found among samples at each locus. Larger kits are more costly (Hutter et al., 2019; Couvreur et al., 2019), and therefore to keep costs manageable universal bait kits balance the number of baits synthesized, and hence bait sequence diversity for each locus, against the total number of RNA baits strictly required to fully capture diversity at each locus. Incomplete representation of sample sequence diversity in the synthesized baits is in part compensated for by the high affinity of the biochemical interaction in the hybridization reaction binding the RNA bait to the DNA target. This high affinity enables successful capture reactions between the RNA baits and the target DNA even in cases where bait and target sequences differ by ~20% (although Johnson et al., 2019 extended this to 30% when designing the Angiosperms353 kit, based on the findings of Liu et al., 2019), and provides a constraint around the minimal sequence diversity required to capture loci across the desired phylogenetic breadth (Mayer et al., 2016; Branstetter et al., 2017; Faircloth, 2017; Couvreur et al., 2019). This is demonstrated by the wide range of flowering plant groups that have successfully utilized the Angiosperms353 kit (Johnson et al., 2019; Van Andel et al., 2019; Larridon et al., 2020; Shee et al., 2020), as well as many other universal bait kits (e.g., flagellate plants [GoFlag; Breinholt et al., 2021], ferns [Wolf et al., 2018], arachnids [Starrett et al., 2017], Cnidaria [Quattrini et al., 2018], and Gastropoda [Teasdale et al., 2016]).

The assembly of raw sequence reads into the desired locus typically follows one of two strategies: (1) de novo assembly of reads and subsequent matching of contigs to target loci, or (2) mapping reads to each locus, followed by de novo assembly of the mapped reads for each locus. Various pipelines are available to perform locus assembly, such as HybPiper (read‐mapping; Johnson et al., 2016), PHYLUCE (de novo assembly; Faircloth, 2016), and SECAPR (both de novo assembly and read‐mapping; Andermann et al., 2018). For any strategy, a file containing the target loci sequences (i.e., the target file) is required. This is typically the same file that was used to design the baits. For universal‐scale kits, this means that closely related reference sequences might not be present in the target file for a given data set. This raises a question: what if the biochemistry of hybrid enrichment enables the successful capture of target loci DNA in vitro, but subsequent bioinformatic processing of raw or assembled data to reconstruct the target locus is inefficient or fails because there is no suitable reference in silico? A mismatch between biochemical locus capture and bioinformatic locus recovery is expected to have a larger impact in broader‐scale universal kits, or groups where suitable reference sequences are lacking, and could influence locus recovery at any phylogenetic level. To investigate the impact of target file sequence diversity on locus recovery, we developed tools to expand the Angiosperms353 target file and compared locus recovery across a range of phylogenetic depths against the default Angiosperms353 file, using HybPiper for locus assembly.

## METHODS

### Generating the mega353 target file

The target file for the Angiosperms353 kit was downloaded from https://github.com/mossmatters/Angiosperms353/blob/master/Angiosperms353_targetSequences.fasta (referred to here as the ‘default353’ target file). To obtain a phylogenetically diverse set of angiosperm sequences from which to recover the Angiosperms353 loci, transcriptomes were downloaded from the 1KP portal (http://www.onekp.com/public_data.html; Carpenter et al., 2019). A maximum of two samples per genus were added, with samples with the largest number of sequences preferentially included. The resulting set included 566 transcriptomes (see https://github.com/chrisjackson‐pellicle/NewTargets/blob/master/filtering_options.csv).

**FIGURE 1:**
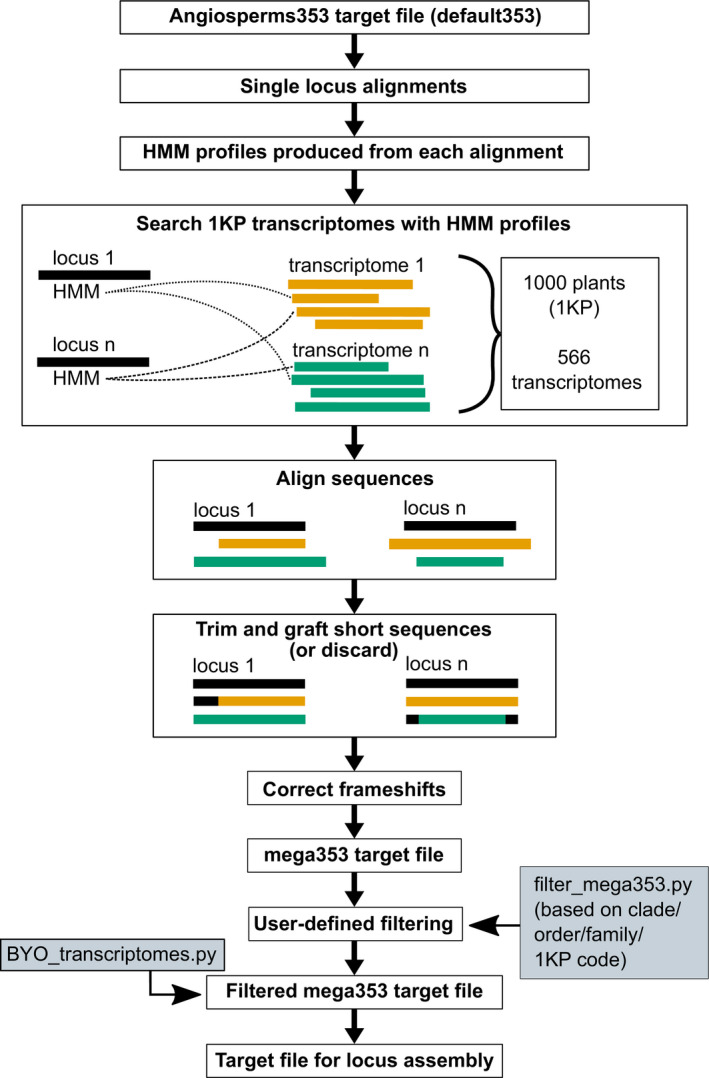
Overview of the steps involved in creating the mega353 target file. First, loci in the default353 file are aligned and hidden Markov model (HMM) profiles are created for each locus. The HMM profiles are used to identify these loci in the 1KP transcriptomes, and transcript hits are added to the alignment. The alignment of each locus is then trimmed, grafted if necessary, a frameshift correction is performed, and all loci are combined in the mega353 target file. The gray boxes indicate steps the user can take to modify the mega353 target file. The mega353 target file can be filtered (based on sample identifiers in the filtering_options.csv file) to select samples included in the target file. The BYO_transcriptome.py script can be used to add GenBank or personal transcriptomes to the filtered mega353 target file.

To create the mega353 target file, the following process was carried out (summarized in Fig. 1). For each locus in the default353 target file, a single locus alignment was produced using MAFFT (Katoh and Standley, 2013), and a corresponding hidden Markov model (HMM) profile was generated using HMMER (Eddy, 2011). HMM profiles were used to search the 1KP transcriptomes using hmmsearch with an *E*‐value cut‐off of 1e‐50, and the top hit (if present) was recovered. Transcriptome hits were added to the corresponding locus alignment, and the 5′ and 3′ termini were trimmed to the longest original target file sequence from either *Arabidopsis thaliana* (L.) Heynh. (Brassicaceae), *Amborella trichopoda* Baill. (Amborellaceae), or *Oryza sativa* L. (Poaceae), as at least one of these three species was included for each locus in the default353 target file. In cases where a transcriptome hit sequence was shorter than the longest original target file sequence for a given locus, the transcriptome hit sequence was extended by grafting with the 5′ and/or 3′ termini of the closest related original sequence. If the resulting grafted sequence was still shorter than the longest original target file sequence, it was grafted again with the longest original sequence. In such cases, the resulting target file sequence was therefore a chimeric construct, and these cases are flagged in the sequence name. This grafting process was necessary as HybPiper translates a single chosen target file sequence for each locus and sample, and the resulting protein sequence is used as a query in Exonerate (Slater and Birney, 2005) to search against assembled nucleotide contigs, using the protein2genome model. Consequently, short protein queries recover truncated nucleotide loci sequences, even if longer contigs have been successfully assembled.

As recovery of target loci using HybPiper requires correct translation of the chosen target file sequences in the first reading frame, any frameshifts observed in trimmed and/or grafted transcriptome hit sequences were corrected or compensated for (see https://github.com/chrisjackson‐pellicle/NewTargets for further details). In cases where a frameshift could not be corrected, the corresponding transcriptome hit sequence was removed for that locus/sample. Finally, sequences were extracted from each locus alignment, gap positions were removed, and all sequences were concatenated to create a new target file.

### Filtering the mega353 target file

To tailor the large mega353 target file to investigation‐specific taxon sampling, we include the script filter_megatarget.py. This script can be used to create a filtered target file based on user‐selected taxa or taxon groups, defined by unique 1KP transcriptome codes, families, orders, or clades (see https://github.com/chrisjackson‐pellicle/NewTargets for full options). In addition to the chosen samples, all sequences from the default353 target file are retained.

### Adding sequences from any transcriptome to any existing target file

As an additional resource, we provide the script BYO_transcriptome.py, which allows sequences from any transcriptome (e.g., from GenBank or personal data) to be added to an existing target file. A target file and a directory of transcriptomes are the only inputs required. For Angiosperms353 analyses, this script can be run using a filtered mega353 target file to expand phylogenetic coverage of target file sequences in a custom manner. The default BYO_transcriptome.py pipeline is the same as that described above for creation of the mega353 target file. Optionally, a flag can be specified to discard short transcriptome hits rather than grafting them, and this option can be applied with a user‐specified length threshold. This allows recovery of non‐chimeric homologous sequences from transcriptomes that can be used in downstream phylogenetic analyses. For additional options, see https://github.com/chrisjackson‐pellicle/NewTargets/wiki.

### Comparing locus recovery between the default353 target file and the expanded mega353 target file

To compare locus recovery between the default353 versus the expanded mega353 target file, we used several data sets, encompassing orders (Asparagales, Sapindales), families (Ericaceae), and genera (*Azorella* Lam., Apiaceae; *Nepenthes* L., Nepenthaceae; *Cyperus* L., Cyperaceae [Larridon et al., 2020]; *Bulbophyllum* Thouars, Orchidaceae), as well as the data set used to test the bait kit in the original Angiosperms353 publication (i.e., the exemplar Angiosperms353 data set; Johnson et al., 2019) (Table 1). A target file corresponding to each data set was produced by filtering the mega353 target file to include sequences for the respective family and/or order, depending on the data set. Because the exemplar Angiosperms353 data set included a phylogenetically diverse set of angiosperms, the full mega353 target file was used without filtering. The filtered Orchidaceae target file was expanded using a set of *Bulbophyllum* transcriptomes and the BYO_transcriptome.py script to create a third, more specific target file for the *Bulbophyllum* data set, in addition to the family and default target files. Trimmomatic (Bolger et al., 2014) was used to trim low‐quality bases and remove Illumina sequencing adapters and primers, with default settings. HybPiper was used to assemble and extract loci, using a nucleotide target file and the flag to call BWA (Li and Durbin, 2009) for each data set, first using the default353 target file as the reference and then the corresponding filtered mega353 target file. For each sample, 16 CPUs and 16 GB of RAM were allocated.

**TABLE 1:** Summary of recovery statistics produced by HybPiper comparing the default353 target set to the mega353 target set (filtered by family or order). Values represent averages of each data set for each target file.

Data set (no. of samples)	Target file	% reads on target (average)	No. of loci with sequences (average)	No. of loci at 75% of target length (average)	Length of concatenated loci (bp, average)
Angiosperms353 exemplar data (41)	default353	22.3%	275.5	117.7	144,283.5
	mega353	32.3%	287.9	132.2	165,867.4
	mega353 vs. default353 % improvement	44.9%	4.5%	12.3%	15%
Asparagales (8)	default353	1.2%	146.9	22.9	55,484.3
	Order (Asparagales)	1.7%	159.5	27.8	65,637.4
	Order vs. default353 % improvement	37.1%	8.6%	21.3%	18.3%
*Azorella* (5)	default353	15.7%	292.8	89	131,292.6
	Family (Apiaceae)	16.3%	299.2	107.6	144,951
	Order (Apiales)	19.4%	309	119.8	158,014.8
	Family vs. default353 % improvement	3.7%	2.2%	20.9%	10.4%
	Order vs. default353 % improvement	23.1%	5.5%	34.6%	20.4%
*Bulbophyllum* (12)	default353	12.30%	238.8	46	93,043
	Family (Orchidaceae)	14.6%	268.2	75.5	122,451.8
	Family + genus (Orchidaceae+*Bulbophyllum*)	15%	273.1	83.8	131,549.8
	Family vs. default353 % improvement	19%	12.3%	64.1%	31.6%
	Family+genus vs. default353 % improvement	22.2%	14.3%	82.2%	41.4%
Cyperaceae (6)	default353	9.4%	201.1667	68	91,865.5
	Family (Cyperaceae)	11.1%	249	103.8333	129,220
	Order (Poales)	12.1%	251.3333	100.3333	131,571
	Family vs. default353 % improvement	18.1%	23.8%	52.7%	40.7%
	Order vs. default353 % improvement	28.3%	24.9%	47.5%	43.2%
Ericaceae (12)	default353	7.5%	307	97.2	145,031.8
	Family (Ericaceae)	11.9%	335.8	185.6	198,784.3
	Order (Ericales)	12.9%	338.6	189.2	205,629
	Family vs. default353 % improvement	60%	9.4%	91%	37.1%
	Order vs. default353 % improvement	73.3%	10.3%	94.7%	41.8%
*Nepenthes* (8)	default353	8.8%	306.6	105.5	145,598.6
	Order (Caryophyllales)	12%	322.9	147.5	182,845.1
	Order vs. default353 % improvement	36%	5.3%	39.78%	25.6%
Sapindales (6)	default353	26.6%	335.6	188.6	193,205.6
	Order (Sapindales)	31.3%	341.4	248.7	229,415.1
	Order vs. default353 % improvement	17.4%	1.7%	31.9%	18.7%
Average percentage improvement		31.9%	10.2%	49.4%	28.7%
Minimum percentage improvement		3.7%	1.7%	12.3%	10.4%
Maximum percentage improvement		73.3%	24.9%	94.7%	43.2%

### Expanding the phylogenetic density of target files for custom bait kits with BYO_transcriptome.py

The input required for the script BYO_transcriptome.py is a target file and a directory of transcriptomes and/or nucleotide sequences corresponding to protein‐coding genes, and it can therefore be used to expand target files from other bait kits. To test this functionality, BYO_transcriptome.py was used to expand target files for an Asteraceae‐specific bait kit (Mandel et al., 2014) and a Hibisceae‐specific bait kit (McLay et al., in prep.).

The Asteraceae bait kit was designed using *Helianthus* *annuus* L. (Asteroideae), *Lactuca sativa* L. (Cichorioideae), and *Carthamus tinctorius* L. (Carduoideae). The Asteraceae target file (comprising only the *H. annuus* and *L. sativa* target sequences) was expanded using 1KP transcriptomes of taxa closely related to Asteraceae tribe Gnaphalieae (Appendix S1). The Hibisceae‐specific bait kit was designed using three Hibisceae transcriptomes, *Abelmoschus esculentus* (L.) Moench, *Hibiscus cannabinus* L., and *Hibiscus syriacus* L. The Hibisceae target file was expanded using available sequence data from the other Malvaceae subfamily Malvoideae tribes, Malveae and Gossypieae (Appendix S2).

## RESULTS

### Sequence number and phylogenetic density in the default353 target file compared to the mega353 target file

**FIGURE 2:**
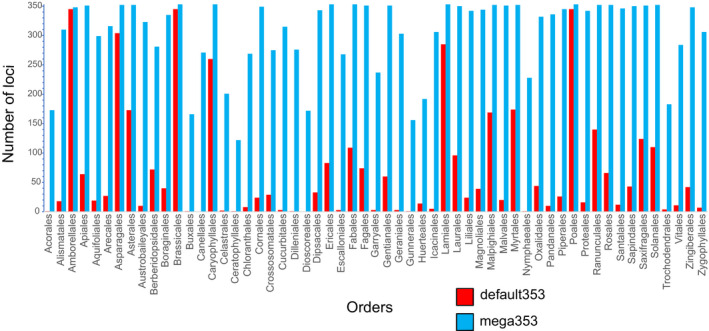
The number of loci represented for each order in the default353 (red) compared to the mega353 (blue) target files.

In the default353 file, there are 4780 target reference sequences, and each locus is represented on average by 13.5 reference sequences (range 6–18). In the mega353 target file, there are 98,994 target reference sequences, and each locus is represented on average by 280 reference sequences (range 17–373). In terms of improvement in phylogenetic density, the default353 target file has an average of 13.5 orders and 13.5 families per locus, whereas the mega353 target file has an average of 49.8 orders and 170 families per locus (see Fig. 2 for order comparisons and Appendix S3 for family comparisons, Appendix S4).

### Comparing locus recovery between the default353 target file and the mega353 target file

To compare locus recovery between the default353 and filtered mega353 target files, results were evaluated using statistics provided by the HybPiper scripts hybpiper_stats.py and get_seq_lengths.py, averaged across all samples for each data set (Table 1). Four statistics were considered: (1) the percentage of reads on target, i.e., the number of reads for a sample that map to the loci in the target file, (2) the number of loci with sequences, or the total number of loci that are in the final locus set for each sample, (3) the number of loci ≥75% of the target length, i.e., of those loci in the final data set, the number that are ≥75% of the length of the target sequence for that loci, and (4) the concatenated length (in base pairs) of the final loci set for each sample.

**FIGURE 3:**
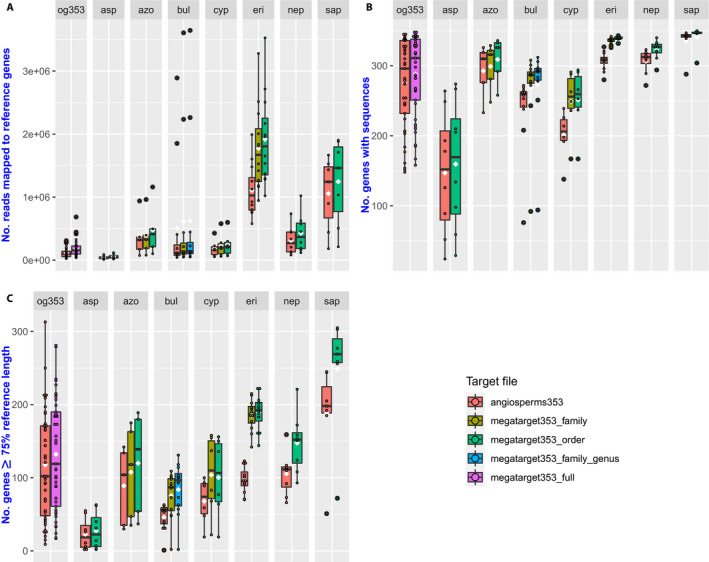
Summary of recovery statistics produced by HybPiper comparing the default353 target set to the mega353 target set (filtered by family or order), for (A) the number of reads mapped to reference sequences, (B) the number of loci with sequences, and (C) the number of loci recovered at ≥75% of reference length. Data set abbreviations for boxplot headings: og353 (Angiosperms353 exemplar), asp (Asparagales), azo (*Azorella*), bul (*Bulbophyllum*), cyp (Cyperaceae), eri (Ericaceae), nep (*Nepenthes*), sap (Sapindales).

**FIGURE 4:**
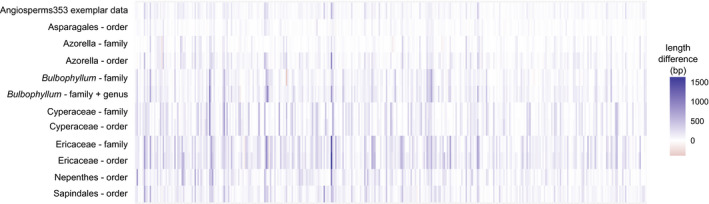
Heatmap of locus length changes for each locus, averaged across all samples for each data set, where the default353 locus lengths are subtracted from the mega353 locus lengths. Increases in length are shown in blue, decreases in length are shown in red.

For each data set, the mega353 target file improved each of these measures (Table 1, Fig. 3). The average percentage of reads on target improved by 31.9% across all data sets (between 3.7% and 73.3%). This had the downstream impact of increasing the number of loci with sequences by an average of 10.2% (24 loci) across all data sets (between 1.7% or six loci, and 24.9% or 50 loci). A greater increase was found in the number of loci at ≥75% of the target length, with an average increase of 49.4% (41 loci) across all data sets (between 12.3% or five loci, and 94.7% or 92 loci). The total length of the concatenated loci increased by an average of 28.7% (from an average of 125 kbp to an average of 155 kbp). This increase in sequence length was an accumulation of length improvements across many loci, rather than large improvements in a small number of loci (Fig. 4). Rarely, locus length decreased for some samples and genes when using the mega353 target file instead of the default353 target file (see Fig. 4 and Appendices [Link], [Link], [Link], [Link], [Link], [Link], [Link], [Link], [Link], [Link], [Link], [Link], red boxes). This most often occurred when many reads mapped to a particular mega353 target sequence reference at some positions, causing HybPiper to select the reference for filtering of assembled contigs via Exonerate. However, this mega353 reference was overall less similar to the sample being assembled than the target reference selected from the default target file. In these instances, HybPiper filtering of assembled contigs via Exonerate returned shorter contig matches (see https://github.com/chrisjackson‐pellicle/NewTargets/wiki for further information). Nonetheless, the overall length of concatenated sequences was still higher when using the mega353‐derived target files compared to the default files.

For the *Bulbophyllum* data set, analyses using the target file with sequences from 12 additional *Bulbophyllum* transcriptomes showed improvements over the filtered Orchidaceae target file, with a 2.5% increase in mapped reads, an 11% increase in loci over 75%, and a 7% increase in concatenated loci length (Table 1).

### Impact of the mega353 target file on HybPiper paralog detection

HybPiper includes a method to detect paralogs as part of its main pipeline, and it alerts the user if at least one paralog is detected for a given sample and locus, in addition to the “primary” contig output. For most of the smaller Angiosperms353 data sets, analyses with the expanded target files resulted in slightly more paralog warnings, with a total increase of only one or two warnings for each data set (Appendix S17). However, for the Sapindales data set, there were an additional 27 paralog warnings. For the custom Asteraceae kit, the expanded target file reduced the total number of paralog warnings by 50.4% (from 698 to 346). For the Hibisceae custom kit, the expanded target file increased the number of paralog warnings by 8.94% (from 705 to 768).

### Impact of the mega353 target file on HybPiper computation time

The first script in the HybPiper pipeline is reads_first.py, which includes mapping of sequence reads to target references and subsequent assembly, and is the most computationally time‐consuming step of the pipeline. For most data sets, using a filtered mega353 target file resulted in a small increase in the number of CPU hours taken by each HybPiper run, because as more reference targets are added the time taken for reads_first.py increases (Appendix S2). However, the CPU hours used by HybPiper to run the Angiosperms353 exemplar data set increased by 40% with the mega353 target file compared to the default353 target file, and by more than 60% for the Ericaceae data set using the order‐filtered mega353 target file.

### Comparing locus recovery for custom bait kits using target files expanded with BYO_transcriptome

Each new target file was compared to its default target file using HybPiper with the approach described above. Seven representative samples from Asteraceae tribe Gnaphalieae, captured using the Asteraceae bait kit (Mandel et al., 2014), were used to compare the default Asteraceae target file (two targets per locus) to the expanded Asteraceae target file (average of 3.88 targets per locus). Five representative taxa from Malvaceae tribes Malveae and Gossypieae, captured using the Hibisceae bait kit, were used to compare the default Hibisceae target file (average of 2.5 targets per locus) to the expanded Malvoideae target file (average 4.34 targets per locus). Locus recovery was improved using the expanded target file for both data sets. This improvement was more pronounced with the expanded Asteraceae target file, with a 31% increase in the number of loci at ≥75% of the target length, and a 22% increase in concatenated loci length (Appendix S18).

## DISCUSSION

We have demonstrated that sequence recovery for a universal sequence capture bait kit can be substantially improved by appropriate tailoring of target files to the group under study. To enable the best possible locus recovery from Angiosperms353 capture data, we have developed an expanded target file using 1KP transcriptomes. As the Angiosperms353 bait kit is becoming increasingly widely used, tools such as those developed here will allow researchers to optimize use of their target enrichment sequence data by assembling more and longer loci, thereby creating larger and more complete data sets for phylogenetic analyses, increasing cost efficiency, and improving data set combinability.

The use of the mega353 target file does increase the computation time for HybPiper. For this reason, we recommend strategically selecting the phylogenetic rank used to filter the target file (i.e., clade, order, or family should be preferred where possible), rather than using the complete mega353 target file. Filtering can be applied using multiple phylogenetic ranks or sample identifiers (see https://github.com/chrisjackson‐pellicle/NewTargets/blob/master/filtering_options.csv). For example, a filtered mega353 target file for Malvales could comprise the target sequences from the order, in addition to selected outgroup sequences (e.g., Brassicaceae), and a specific 1KP sample name (e.g., UPZX, *Cleome gynandra* L., Cleomaceae). Filtering of the mega353 target file allows the user to develop a data set–appropriate target file and ensures a more efficient trade‐off between increased locus recovery and computational time.

We have demonstrated these improvements using the bioinformatic tool HybPiper. However, it is likely that any pipeline that involves read‐mapping to reference files as a first step will see similar improvements when provided with a more phylogenetically dense target file (e.g., tools such as HybPhyloMaker [Fér and Schmickl, 2018]). It is less clear whether expanded target files would improve locus recovery using tools that perform a de novo assembly first, and then map contigs to a reference sequence (such as SECAPR [Andermann et al., 2018] or PHYLUCE [Faircloth, 2016]). Regardless, HybPiper is currently the most widely used tool to assemble target enrichment data sets in plants, and researchers using HybPiper can obtain improved locus recovery using expanded target files.

Finally, our BYO_transcriptome.py script can be used to incorporate additional target sequences from any available transcriptome, and we have shown that this tool can be used with target files from custom bait kits to improve locus recovery. With the growing number of transcriptomes and whole genome data becoming available in public repositories, the approach developed here will prove to be an increasingly valuable resource for efficient recovery of target enrichment data.

## AUTHOR CONTRIBUTIONS

T.G.B.M. and C.J.J. conceived and developed the bioinformatic workflow and drafted the manuscript; T.G.B.M., C.J.J., A.N.S.L., and L.S. performed data analyses​; and all authors contributed data and approved the final manuscript.

## Supporting information


**APPENDIX S1**. Samples used to expand the custom bait kit target files using ‘BYO_transcriptomes.py’.

**APPENDIX S2**. CPU hours used by the HybPiper pipeline to complete for each data set and each target file. HybPiper was allocated 16 CPUs and 16 GB of RAM for each data set.

**APPENDIX S3**. The number of loci represented for each family in the default353 (red) compared to the mega353 (blue) target files.

**APPENDIX S4**. The number of target sequences in the default353 target file compared to the mega353 target file, including the average number of targets per locus, and the average number of orders and families for each locus.

**APPENDIX S5**. Heatmap of locus lengths for each sample for each locus for the Angiosperms353 exemplar data set, where the default353 locus lengths are subtracted from the mega353 locus lengths. Increases in length are shown in blue; decreases in length are shown in red.

**APPENDIX S6**. Heatmap of locus lengths for each sample for each locus for the Asparagales data set, where the default353 locus lengths are subtracted from the mega353 (order filtered) locus lengths. Increases in length are shown in blue; decreases in length are shown in red.

**APPENDIX S7**. Heatmap of locus lengths for each sample for each locus for the *Azorella* data set, where the default353 locus lengths are subtracted from the mega353 (family filtered) locus lengths. Increases in length are shown in blue; decreases in length are shown in red.

**APPENDIX S8**. Heatmap of locus lengths for each sample for each locus for the *Azorella* data set, where the default353 locus lengths are subtracted from the mega353 (order filtered) locus lengths. Increases in length are shown in blue; decreases in length are shown in red.

**APPENDIX S9**. Heatmap of locus lengths for each sample for each locus for the *Bulbophyllum* data set, where the default353 locus lengths are subtracted from the mega353 (family filtered) locus lengths. Increases in length are shown in blue; decreases in length are shown in red.

**APPENDIX S10**. Heatmap of locus lengths for each sample for each locus for the *Bulbophyllum* data set, where the default353 locus lengths are subtracted from the mega353 (family + genus filtered) locus lengths. Increases in length are shown in blue; decreases in length are shown in red.

**APPENDIX S11**. Heatmap of locus lengths for each sample for each locus for the Cyperaceae data set, where the default353 locus lengths are subtracted from the mega353 (family filtered) locus lengths. Increases in length are shown in blue; decreases in length are shown in red.

**APPENDIX S12**. Heatmap of locus lengths for each sample for each locus for the Cyperaceae data set, where the default353 locus lengths are subtracted from the mega353 (order filtered) locus lengths. Increases in length are shown in blue; decreases in length are shown in red.

**APPENDIX S13**. Heatmap of locus lengths for each sample for each locus for the Ericaceae data set, where the default353 locus lengths are subtracted from the mega353 (family filtered) locus lengths. Increases in length are shown in blue; decreases in length are shown in red.

**APPENDIX S14**. Heatmap of locus lengths for each sample for each locus for the Ericaceae data set, where the default353 locus lengths are subtracted from the mega353 (order filtered) locus lengths. Increases in length are shown in blue; decreases in length are shown in red.

**APPENDIX S15**. Heatmap of locus lengths for each sample for each locus for the *Nepenthes* data set, where the default353 locus lengths are subtracted from the mega353 (order filtered) locus lengths. Increases in length are shown in blue; decreases in length are shown in red.

**APPENDIX S16**. Heatmap of locus lengths for each sample for each locus for the Sapindales data set, where the default353 locus lengths are subtracted from the mega353 (order filtered) locus lengths. Increases in length are shown in blue; decreases in length are shown in red.

**APPENDIX S17**. Summary of paralog warnings produced by HybPiper for the default353 and mega353 target files.

**APPENDIX S18**. Comparing custom bait kit target files (Asteraceae/Hibisceae) that were expanded using BYO_transcriptomes.py. Values represent averages of each data set for each target file.

## Data Availability

NewTargets is an open source software that is freely available on GitHub (https://github.com/chrisjackson‐pellicle/NewTargets) for Linux or OSX under the GNU General Public License v3. NewTargets is written in Python and requires Python 3.7 or higher. Documentation for the software can be found on GitHub (https://github.com/chrisjackson‐pellicle/NewTargets/wiki). Sources of the publicly available data sets used in this study include the 1KP project (http://www.onekp.com/samples/list.php), the original Angiosperms353 sequence reads (National Center for Biotechnology Information Sequence Read Archive [SRA]: SRP151601), the Cyperaceae data set (SRA BioProject PRJNA553989), the *Nepenthes* data set (SRA BioProject PRJEB35235), and the Sapindales data set (European Nucleotide Archive project PRJEB35285, from the Plant and Fungal Trees of Life [PAFTOL] project).
