# Supplementary material for: New targets acquired: Improving locus recovery from the Angiosperms353 probe set
Source: Appl Plant Sci. 2021 Jun 14;9(7):10.1002/aps3.11420. doi: 10.1002/aps3.11420 (PMC8312740; doi:10.1002/aps3.11420)
Supplement: Supplementary file 1 — APPENDIX S1. Samples used to expand the custom bait kit target files using ‘BYO_transcriptomes.py’. [file APS3-9--s017.docx]

**APPENDIX S1.** Samples used to expand the custom bait kit target files using ‘BYO_transcriptomes.py’.

| **Bait kit data set** | **Samples added (source)** |
| --- | --- |
| Asteraceae – Mandel et al., 2014 | *Senecio rowleyanus* H. Jacobsen (1KP BMSE)  *Leontopodium alpinum* (Ten.) A. Huet ex Hand.-Mazz. (1KP DOVJ)  *Matricaria matricarioides* (Less.) Porter (1KP OAGK)  *Solidago canadensis* L. (1KP TEZA) |
| Hibisceae – McLay et al., in prep. | *Hoheria angustifolia* Raoul (1KP ZSAB)  *Gossypium australe* F. Muell. (GenBank PRJNA513946) |
