# Supplementary material for: New targets acquired: Improving locus recovery from the Angiosperms353 probe set
Source: Appl Plant Sci. 2021 Jun 14;9(7):10.1002/aps3.11420. doi: 10.1002/aps3.11420 (PMC8312740; doi:10.1002/aps3.11420)
Supplement: Supplementary file 2 — APPENDIX S2. CPU hours used by the HybPiper pipeline to complete for each data set and each target file. HybPiper was allocated 16 CPUs and 16 GB of RAM for each data set. [file APS3-9--s016.docx]

**APPENDIX S2.** CPU hours used by the HybPiper pipeline to complete for each data set and each target file. HybPiper was allocated 16 CPUs and 16 GB of RAM for each data set.

| **Data set** | **Target file** | **CPU hours** |
| --- | --- | --- |
| Angiosperm353 exemplar data | default353 | 111.8 |
|  | mega353 | 156.7 |
| Asparagales | default353 | 12.6 |
|  | Order | 14.3 |
| *Azorella* | default353 | 102.4 |
|  | Family | 110.4 |
|  | Order | 123.6 |
| Cyperaceae | default353 | 10.2 |
|  | Family | 11.7 |
|  | Order | 12.7 |
| Ericaceae | default353 | 177.6 |
|  | Family | 267.1 |
|  | Order | 292.3 |
| Nepenthes | default353 | 36.7 |
|  | Order | 47.5 |
| Sapindales | default353 | 81.5 |
|  | Order | 87.8 |
