# Supplementary material for: New targets acquired: Improving locus recovery from the Angiosperms353 probe set
Source: Appl Plant Sci. 2021 Jun 14;9(7):10.1002/aps3.11420. doi: 10.1002/aps3.11420 (PMC8312740; doi:10.1002/aps3.11420)

McLay et al.—Applications in Plant Sciences 2021 9(7)—Data Supplement S3. Page 1 of 1.
DOI 10.1002/aps3.11420

APPENDIX S3. The number of loci represented for each family in the default353 (red) compared to the mega353 (blue) target files.

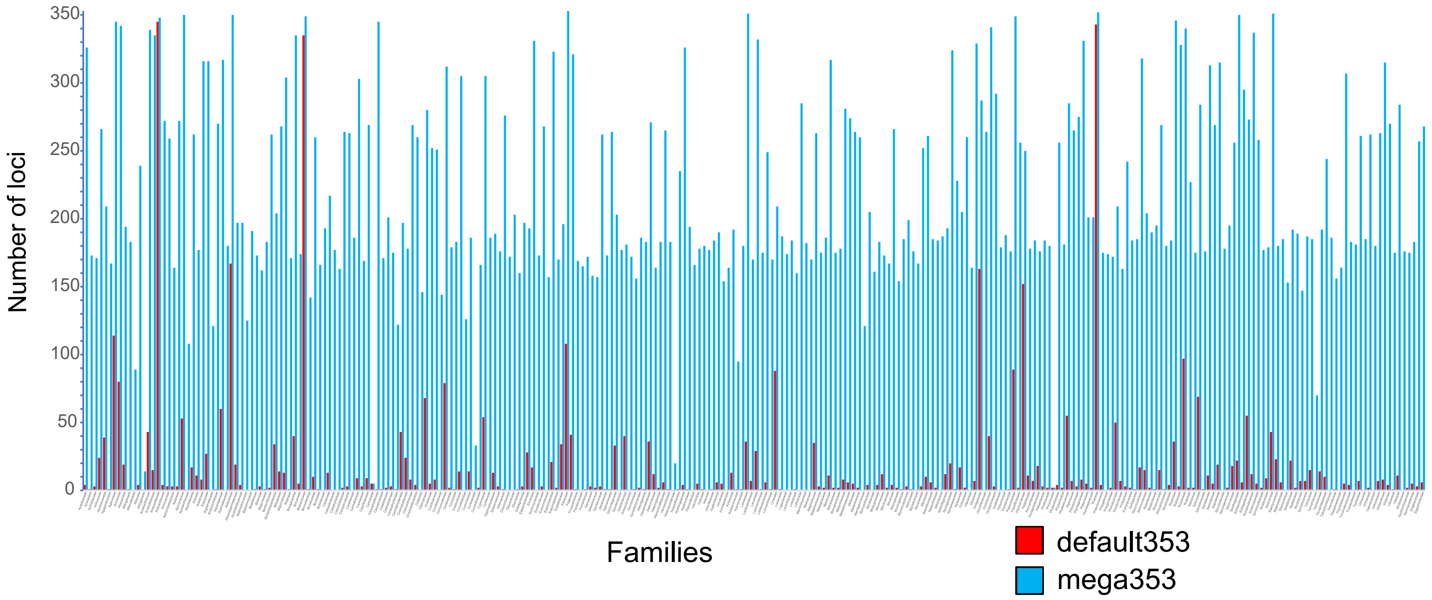

Supplement: Supplementary file 3 — APPENDIX S3. The number of loci represented for each family in the default353 (red) compared to the mega353 (blue) target files. [file APS3-9--s002.pdf]
