# Supplementary material for: New targets acquired: Improving locus recovery from the Angiosperms353 probe set
Source: Appl Plant Sci. 2021 Jun 14;9(7):10.1002/aps3.11420. doi: 10.1002/aps3.11420 (PMC8312740; doi:10.1002/aps3.11420)
Supplement: Supplementary file 4 — APPENDIX S4. The number of target sequences in the default353 target file compared to the mega353 target file, including the average number of targets per locus, and the average number of orders and families for each locus. [file APS3-9--s001.docx]

**APPENDIX S4**. The number of target sequences in the default353 target file compared to the mega353 target file, including the average number of targets per locus, and the average number of orders and families for each locus.

| **Target file metric** | **default353** | **mega353** |
| --- | --- | --- |
| Total number of target sequences | 4780 | 98994 |
| Average number of target reference sequences per locus  (minimum ­­­­– maximum) | 13.5  (6–18) | 280  (17–373) |
| Average number of orders for each locus  (minimum – maximum, total) | 13.5  (6–18, 55) | 49.8  (13–57, 57) |
| Average number of families for each locus (minimum – maximum, total) | 13.5  (6–18, 226) | 170  (14–214, 276) |
