# Supplementary material for: New targets acquired: Improving locus recovery from the Angiosperms353 probe set
Source: Appl Plant Sci. 2021 Jun 14;9(7):10.1002/aps3.11420. doi: 10.1002/aps3.11420 (PMC8312740; doi:10.1002/aps3.11420)

McLay et al.—Applications in Plant Sciences 2021 9(7)—Data Supplement S8. Page 1 of 1.
DOI 10.1002/aps3.11420

APPENDIX S8. Heatmap of locus lengths for each sample for each locus for the *Azorella* data set, where the default353 locus lengths are subtracted from the mega353 (order filtered) locus lengths. Increases in length are shown in blue; decreases in length are shown in red.

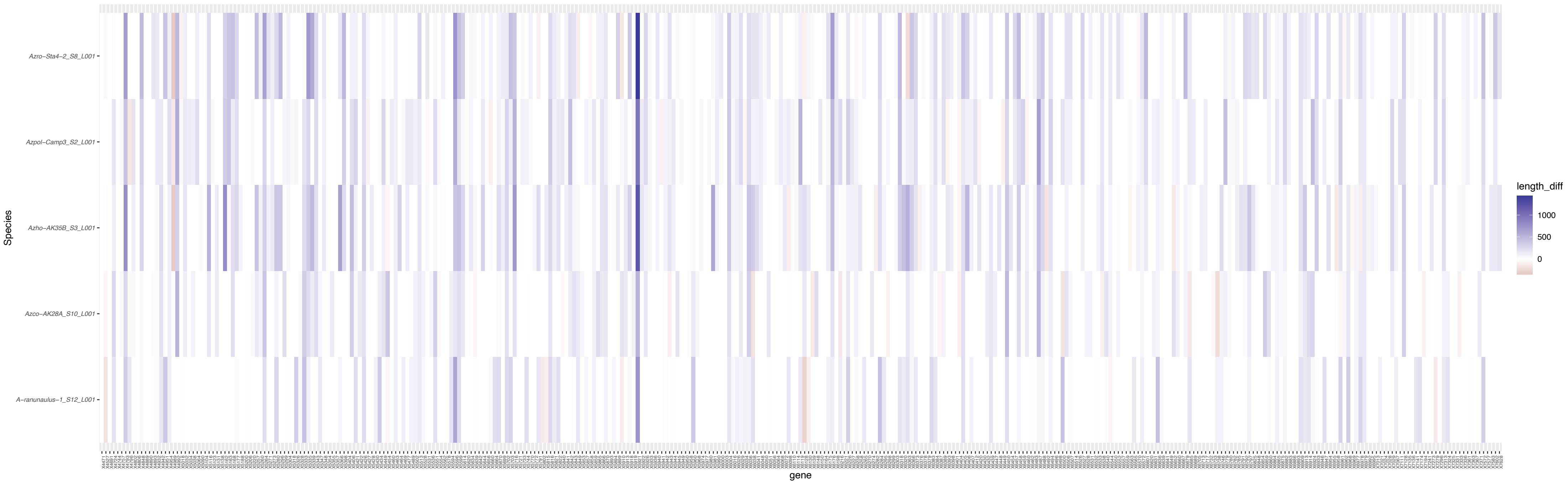

Supplement: Supplementary file 8 — APPENDIX S8. Heatmap of locus lengths for each sample for each locus for the Azorella data set, where the default353 locus lengths are subtracted from the mega353 (order filtered) locus lengths. Increases in length are shown in blue; decreases in length are shown in red. [file APS3-9--s005.pdf]
