# Supplementary material for: New targets acquired: Improving locus recovery from the Angiosperms353 probe set
Source: Appl Plant Sci. 2021 Jun 14;9(7):10.1002/aps3.11420. doi: 10.1002/aps3.11420 (PMC8312740; doi:10.1002/aps3.11420)

DOI 10.1002/aps3.11420

**APPENDIX S10.** Heatmap of locus lengths for each sample for each locus for the *Bulbophyllum* data set, where the default353 locus lengths are subtracted from the mega353 (family + genus filtered) locus lengths. Increases in length are shown in blue; decreases in length are shown in red.

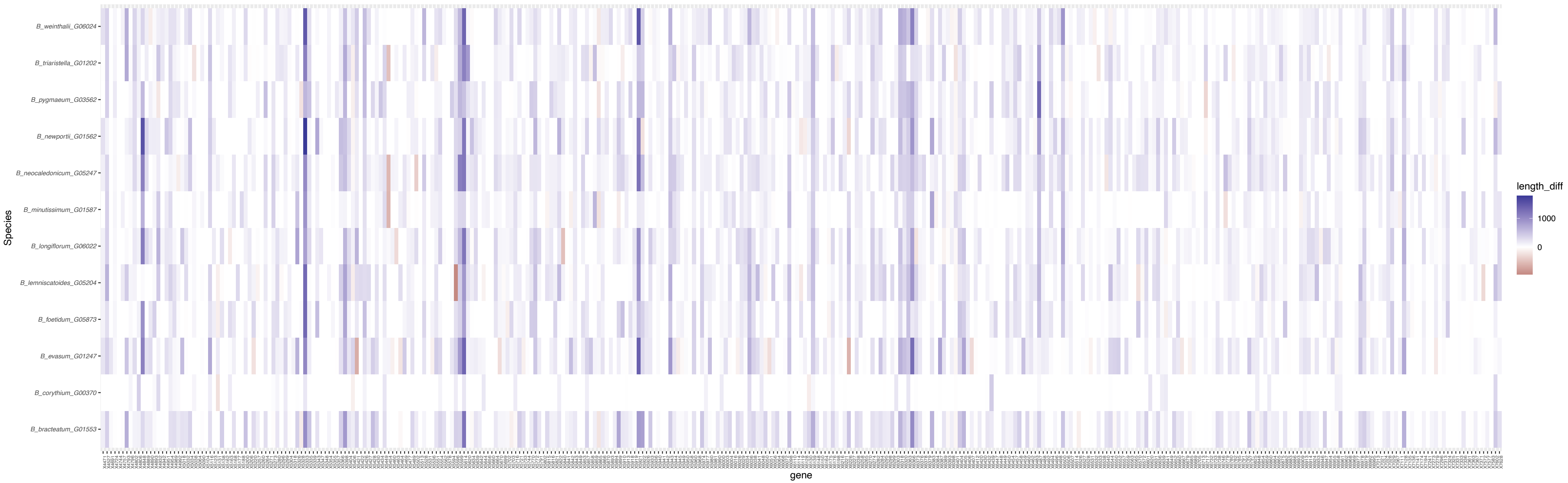

Supplement: Supplementary file 10 — APPENDIX S10. Heatmap of locus lengths for each sample for each locus for the Bulbophyllum data set, where the default353 locus lengths are subtracted from the mega353 (family + genus filtered) locus lengths. Increases in length are shown in blue; decreases in length are shown in red. [file APS3-9--s007.pdf]
