# Supplementary material for: New targets acquired: Improving locus recovery from the Angiosperms353 probe set
Source: Appl Plant Sci. 2021 Jun 14;9(7):10.1002/aps3.11420. doi: 10.1002/aps3.11420 (PMC8312740; doi:10.1002/aps3.11420)

DOI 10.1002/aps3.11420

APPENDIX S15. Heatmap of locus lengths for each sample for each locus for the *Nepenthes* data set, where the default353 locus lengths are subtracted from the mega353 (order filtered) locus lengths. Increases in length are shown in blue; decreases in length are shown in red.

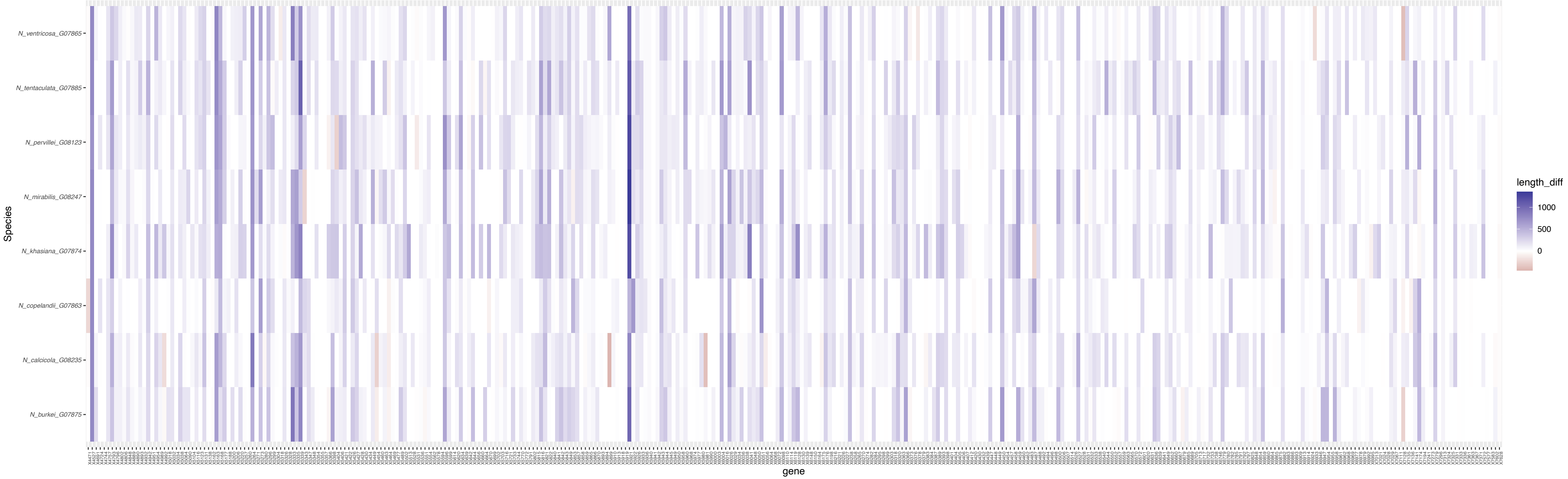

Supplement: Supplementary file 15 — APPENDIX S15. Heatmap of locus lengths for each sample for each locus for the Nepenthes data set, where the default353 locus lengths are subtracted from the mega353 (order filtered) locus lengths. Increases in length are shown in blue; decreases in length are shown in red. [file APS3-9--s012.pdf]
