# Supplementary material for: New targets acquired: Improving locus recovery from the Angiosperms353 probe set
Source: Appl Plant Sci. 2021 Jun 14;9(7):10.1002/aps3.11420. doi: 10.1002/aps3.11420 (PMC8312740; doi:10.1002/aps3.11420)
Supplement: Supplementary file 17 — APPENDIX S17. Summary of paralog warnings produced by HybPiper for the default353 and mega353 target files. [file APS3-9--s014.docx]

**APPENDIX S17.** Summary of paralog warnings produced by HybPiper for the default353 and mega353 target files.

| **Data set**  **(no. of samples)** | **Target file** | **Total no. of paralog warnings** | **Average no. of paralog warnings per sample** |
| --- | --- | --- | --- |
| Angiosperm353 exemplar data (43) | default353 | 244 | 5.95 |
|  | mega353 | 248 | 6.05 |
|  |  |  |  |
|  | mega353 vs. default353 % improvement | 1.6% |  |
| Asparagales (8) | default353 | 2 | 0.25 |
|  | Order (Asparagales) | 3 | 0.38 |
|  |  |  |  |
|  | Order vs. default353 % improvement | 50% |  |
| *Azorella* (5) | default353 | 23 | 4.6 |
|  | Family (Apiaceae) | 21 | 4.2 |
|  | Order (Apiales) | 19 | 3.8 |
|  |  |  |  |
|  | Family vs. default353 % improvement | −9% |  |
|  | Order vs. default353 % improvement | −17% |  |
| *Bulbophyllum* (12) | default353 | 3 | 1 |
|  | Family (Orchidaceae) | 4 | 1.3 |
|  | Family + genus (Orchidaceae+*Bulbophyllum*) | 6 | 2 |
|  |  |  |  |
|  | Family vs. default353 % improvement | 33.3% |  |
|  | Family+genus vs. default353 % improvement | 100% |  |
| Cyperaceae (6) | default353 | 6 | 1 |
|  | Family (Cyperaceae) | 10 | 1.7 |
|  | Order (Poales) | 10 | 1.7 |
|  |  |  |  |
|  | Family vs. default353 % improvement | 66.7% |  |
|  | Order vs. default353 % improvement | 66.7% |  |
| Ericaceae (12) | default353 | 0 | 0 |
|  | Family (Ericaceae) | 1 | 0.08 |
|  | Order (Ericales) | 1 | 0.08 |
|  |  |  |  |
|  | Family vs. default353 % improvement | 0% |  |
|  | Order vs. default353 % improvement | 0% |  |
| *Nepenthes* (8) | default353 | 2 | 0.25 |
|  | Order (Caryophyllales) | 3 | 0.375 |
|  |  |  |  |
|  | Order vs. default353 % improvement | 50% |  |
| Sapindales (6) | default353 | 90 | 12.86 |
|  | Order (Sapindales) | 117 | 16.7 |
|  |  |  |  |
|  | Order vs. default353 % improvement | 30% |  |
| \| Asteraceae custom kit (6) \| \| --- \| \|  \| \|  \| \|  \| | default | 698 | 116.3 |
|  | BYO transcriptome expanded | 346 | 57.7 |
|  |  |  |  |
|  | BYO expanded-default % improvement | −50.4% |  |
| \| Malvaceae custom kit (7) \| \| --- \| \|  \| \|  \| \|  \| | default | 705 | 100.7 |
|  | BYO transcriptome expanded | 768 | 109.7 |
|  |  |  |  |
|  | BYO expanded-default % improvement | 8.94% |  |
