# Supplementary material for: New targets acquired: Improving locus recovery from the Angiosperms353 probe set
Source: Appl Plant Sci. 2021 Jun 14;9(7):10.1002/aps3.11420. doi: 10.1002/aps3.11420 (PMC8312740; doi:10.1002/aps3.11420)
Supplement: Supplementary file 18 — APPENDIX S18. Comparing custom bait kit target files (Asteraceae/Hibisceae) that were expanded using BYO_transcriptomes.py. Values represent averages of each data set for each target file. [file APS3-9--s015.docx]

**APPENDIX S18.** Comparing custom bait kit target files (Asteraceae/Hibisceae) that were expanded using BYO_transcriptomes.py. Values represent averages of each data set for each target file.

| **Bait kit data set**  **(no. of samples tested)** | **Target file** | **Percentage of reads on target**  **(average)** | **No. of loci with sequences**  **(average)** | **No. of loci at 75% of target length**  **(average)** | **Length of concatenated loci (bp, average)** |
| --- | --- | --- | --- | --- | --- |
| Asteraceae – Mandel et al., 2014 (7) | Asteraceae target file | 15% | 560.2 | 332.7 | 158,938.5 |
|  | Expanded target file | 23% | 665.5 | 435.8 | 194,377 |
|  | % improvement | 54.3% | 18.8% | 31% | 22.3% |
| Hibisceae – McLay et al., in prep.  (5) | Hibisceae target file | 24.8% | 504.8 | 449.6 | 247,443.6 |
|  | Expanded target file | 27.8% | 512.6 | 461.6 | 267,151.8 |
|  | % improvement | 12.4% | 1.6% | 2.7% | 8% |
